# Nanopore sequencing reveals genomic map of CTX-M-type extended-spectrum β-lactamases carried by *Escherichia coli* strains isolated from blue mussels (*Mytilus edulis*) in Norway

**DOI:** 10.1186/s12866-020-01821-8

**Published:** 2020-05-25

**Authors:** Didrik H. Grevskott, Francisco Salvà-Serra, Edward R. B. Moore, Nachiket P. Marathe

**Affiliations:** 1 Department of Contaminants and Biohazards, Institute of Marine Research (IMR), Bergen, Norway; 2 Department of Infectious Diseases, Institute of Biomedicine, Sahlgrenska Academy, University of Gothenburg, Gothenburg, Sweden; 3 Culture Collection University of Gothenburg (CCUG), Sahlgrenska Academy, Gothenburg, Sweden; 4 Centre for Antibiotic Resistance Research (CARe), University of Gothenburg, Gothenburg, Sweden; 5 Department of Clinical Microbiology, Sahlgrenska University Hospital, Region Västra Götaland, Gothenburg, Sweden; 6 Microbiology, Department of Biology, University of the Balearic Islands, Palma de Mallorca, Spain

**Keywords:** Norway, *Escherichia coli*, ESBL, Nanopore, Genome sequence, MinION, Long-read sequencing, Antibiotic resistance

## Abstract

**Background:**

Environmental surveillance of antibiotic resistance can contribute towards better understanding and management of human and environmental health. This study applied a combination of long-read Oxford Nanopore MinION and short-read Illumina MiSeq-based sequencing to obtain closed complete genome sequences of two CTX-M-producing multidrug-resistant *Escherichia coli* strains isolated from blue mussels (*Mytilus edulis*) in Norway, in order to understand the potential for mobility of the detected antibiotic resistance genes (ARGs).

**Results:**

The complete genome sequence of strain 631 (*E. coli* sequence type 38) was assembled into a circular chromosome of 5.19 Mb and five plasmids (between 98 kb and 5 kb). The majority of ARGs cluster in close proximity to each other on the chromosome within two separate multidrug-resistance determining regions (MDRs), each flanked by IS*26* transposases. MDR-1 carries *bla*_TEM-1_, *tmrB*, *aac(3)-IId*, *aadA5*, *mph(A)*, *mrx*, *sul1*, *qacE*Δ1 and *dfrA17*; while MDR-2 harbors *aph(3″)-Ib*, *aph(6)-Id*, *bla*_TEM-1_, *catA1*, *tet(D)* and *sul2*. Four identical chromosomal copies of *bla*_CTX-M-14_ are located outside these regions, flanked by IS*Ec9* transposases. Strain 1500 (*E. coli* sequence type 191) exhibited a circular chromosome of 4.73 Mb and two plasmids (91 kb and 4 kb). The 91 kb conjugative plasmid belonging to IncI1 group carries *bla*_CTX-M-15_ and *bla*_TEM-1_ genes.

**Conclusion:**

This study confirms the efficacy of combining Nanopore long-read and Illumina short-read sequencing for determining complete bacterial genome sequences, enabling detection and characterization of clinically important ARGs in the marine environment in Norway, with potential for further dissemination. It also highlights the need for environmental surveillance of antibiotic resistance in low prevalence settings like Norway.

## Background

Extended-spectrum β-lactamase (ESBL)-producing *Enterobacteriaceae* represent an emerging public health threat, for which research and urgent development of new antibiotics is needed [1]. Extended-spectrum β-lactamases are a group of enzymes that hydrolyze β-lactam antibiotics, including 3^rd^ generation cephalosporins [2]. These enzymes are divided into molecular class A, C and D, based on the protein sequences [3]. Among ESBLs, plasmid-mediated class A β-lactamases belonging to the CTX-M-type are prominent ESBLs in the clinics, especially in Europe [4, 5]. CTX-M-producing *Escherichia coli* are dominated by a few high-risk clones, such as sequence type (ST) 131 and ST38 [6, 7]. *E. coli* ST131 and ST38 are recognized as enteroaggregative *E. coli* (EAEC) that can also cause extra-intestinal infections, including blood stream infection and urinary tract infection [8–10].

Environmental niches, including the aquatic environment, serve as a source of and/or a dissemination route for antibiotic resistance genes (ARGs) and resistant bacteria [11–14]. Clinically relevant ARGs and pathogens are introduced into the environment via different routes, such as through sewage contamination [15], waste from livestock production [16] and runoff from land [17]. Once introduced into the environment, ARGs and pathogens interact with environmental bacteria when sharing, at least temporarily, the same habitats [18]. Proximity and interactions within environmental niches provide opportunities for acquisition of resistance genes via horizontal transfer [18, 19]. Moreover, environmental pollution with antibiotics and other antimicrobial substances lead to selection of ARGs and resistant bacteria [20, 21]. Such environments, thus, may be hotspots for further dissemination of ARGs and resistant bacterial strains.

The southern and eastern countries in Europe present high-risk of antimicrobial resistance (AMR) due to, in part, extensive use of antibiotics [22, 23]. For instance, the prevalence of invasive *E. coli* isolates resistant to 3^rd^ generation cephalosporins was 29.5% in Italy, in 2017 [22]. Accordingly, the prevalence of AMR in the environment was high [24], e.g., 15% of the *E. coli* strains (*n* = 141) isolated from Venus clams (*Chamelea gallina*) in Italy carried ESBLs [25]. In contrast, Norway represents a low prevalence setting, in terms of antibiotic use [23] and prevalence of AMR [22]. The prevalence of ESBL-positive *E. coli* in Norway was 6.6 and 3.0% from blood and urine, respectively, in 2017 [26]. Although there is limited knowledge, the overall prevalence of AMR in the environment in Norway is low. In a previous study, we detected only two ESBL-positive *E. coli* strains (out of 199 analyzed), isolated from blue mussels (*Mytilus edulis*) in Norway [27].

With the advent of next-generation sequencing, whole-genome sequencing is increasingly used for resolving questions of bacterial taxonomy as well as for studying the genetic contents of particular strains [28]. Short-read sequencing technologies, such as Illumina and Ion Torrent, allow fragmented genome assembly, i.e., draft genome and, occasionally, complete closed genome sequences [29, 30]. Draft genome sequences are suitable for detecting genes present in a given strain and for basic characterization and phylogenetic studies [31]. However, draft genome sequences do not reveal the complete metabolic potential of the given strains. Long-read sequencing technologies, such as Oxford Nanopore and PacBio, allow assembly of complete genome sequences [32, 33], including the sequences of associated plasmids, which often carry metabolic genes and ARGs. However, owing to higher sequencing error rates associated with the long-read sequencing technologies, hybrid assembly using a combination of low-error short-reads as well as the long-reads, has been successfully applied to obtain reliable, complete closed genome sequences of bacterial strains [34].

The aim of this study was to apply a combination of long-read Nanopore and short-read Illumina-based sequencing to obtain high-quality complete genome sequences of the two ESBL-positive *E. coli* strains (631 and 1500) isolated from blue mussels (*M. edulis*) collected from coastal waters in Norway [27], in order to determine the genomic map of resistance genes and their potential for horizontal transfer.

## Results

### Complete genome sequences of the two CTX-M-producing *E. coli* strains

The Oxford Nanopore sequencing run generated 471,175 sequence reads for strain 631 and 576,474 sequence reads for strain 1500, with average read length of 7.7 kb and 6.7 kb, respectively. The longest read for strain 631 was 105,952 bp and for strain 1500 was 125,266 bp. The average Phred quality score of the raw reads for Nanopore was 10.0 for both the strains (i.e., probability of error 0.1). The Nanopore-solo sequence assembly yielded six contigs for strain 631 and three contigs for strain 1500. The Illumina sequencing of strains 631 and 1500 generated 1,362,720 and 2,769,670 paired-end reads, respectively. After quality trimming, the average length of the reads was 227 bp for strain 631 and 211 bp for strain 1500. The longest read was 251 bp for both the strains. For Illumina reads, the average Phred quality scores of the trimmed reads were 34.5 for strain 631 and 34.9 for strain 1500 (i.e., probability of error < 0.001). The assembly of Illumina-solo sequences produced 102 and 50 contigs (> 500 bp) for strains 631 and 1500, respectively.

In order to obtain highly accurate closed complete genome sequences of strains 631 and 1500, hybrid *de novo* assembly of Nanopore long-reads and Illumina short-reads was performed for each strain. The complete genome of strain 631 (GenBank accession number: CP040263-CP040268) was assembled into six contigs; one contig representing a complete circular chromosome of 5,191,486 bp and five plasmids, ranging from 97,726 bp to 5165 bp (Table 1). All ARGs, virulence genes (except for the *espI* gene detected on plasmid pEc631_1) and biocide/metal resistance genes (BMRGs) were located on the chromosome of this strain. Strain 1500 (GenBank accession number: CP040269-CP040271) exhibits a circular chromosome of 4,736,377 bp and two plasmids of 91,123 bp and 4087 bp (Table 1). This strain carries all virulence genes and BMRGs on the chromosome. However, β-lactamase genes *bla*_CTX-M-15_ and *bla*_TEM-1_ are located on the plasmid pEc1500_CTX. Genome assembly statistics and complete overview of the genome sequences of strains 631 and 1500 are presented in Additional files 1 and 2, respectively. Additionally, a list of the virulence genes and BMRGs detected in strains 631 and 1500 (i.e., gene names and their function) are presented in Additional file 3. Conjugal transfer genes detected by searching through the GenBank files of the annotated genome sequences of strains 631 and 1500 are listed in Additional file 4.

**Table 1:** Overview of antibiotic resistance genes, virulence genes, biocide/metal resistance genes and conjugal transfer genes detected in *Escherichia coli* strains 631 (GenBank accession number: CP040263-CP040268) and 1500 (GenBank accession number: CP040269-CP040271) complete genome sequences

Strain	Contig	Size (bp)	Plasmid type	Antibiotic resistance genes	Virulence genes^**α**^	Biocide/metal resistance genes^**β**^	Conjugal transfer genes^**γ**^
631	Chromosome	5,191,486	–	*aac(3)-IId, aadA5, aph(3″)-Ib, aph(6)-Id, bla*_CTX-M-14_*, bla*_TEM-1_, *catA1, dfrA17, mph(A), mrx, qacE*Δ1*, sul1, sul2, tet(D), tmrB*	*ecpA-E, ecpR, elfA/G, elfC-D, eaeH, hcpA-C, papX, fimA-I, cah, ehaB, air/eaeX, upaG/ehaG, upaH, ibeB-C, tia, chuA, chuS-U, chuW-Y, sitA-D, fyuA, irp1–2, ybtA/E/X, ybtP-Q, ybtS-U, espL1/L4/R1/X1, espX4–5, espY1–4,* ACE T6SS-like gene*, aec11, aec15–19, aec22–32,* two SCI-I T6SS-like genes, *hlyE/clyA*	*acrA-B, arsB-C, arsR, asr, baeR-S, bcr, chrA copA, corA-D, cpxA/R, cueO, cusB/S, cutA/C, dsbA-C, emrA-B, emrD/K/R/Y, nikA-E, nikR, sodA-B, soxR-S, modA-C, modE, evgA/S, gadA-B, gadX, ibpA-B, marA/R, pstA-C, pstS, tehA-B, ybtP-Q, ydeI, ydeO-P, fabI, glpF, iclR, mgtA, mntR, nfsA, oxyRkp, phoB, pitA, robA, rpoS, sugE, tolC, ychH, ygiW, yhcN, yieF, yodD, yqjH, acrD/yffA, acrE/envC, acrF/envD, actP/yjcG, bhsA/ycfR/comC, comR/ycfQ, cueR/ybbI, cusA/ybdE, cusC/ylcB, cusF/cusX, cusR/ylcA, cutE/lnt, cutF/nlpE, emrE/mvrC, fetA/ybbL, fetB/ybbM, fieF/yiip, gadC/xasA, gadE/yhiE, gadW/yhiW, hdeA/yhiB, hdeB/yhiC, mdfA/cmr, mdtA/yegM, mdtB/yegN, mdtC/yegO, mdtE/yhiU, mdtF/yhiV, mdtG/yceE, mdtI/ydgE, mdtJ/ebrB/ydgF, mdtK/ydhE, mdtN/yjcR, mntH/yfeP, mntP/yebN, ostA/lptD, rcnA/yohM, rcnB/yohN, rcnR/yohL, ymgB/ariR, zinT/yodA, zitB/ybgR, zntA/yhhO, zntR/yhdM, znuA/yebL, znuB/yebI, znuC/yebM, zraR/hydH, zraS/hydG, zupT/ygiE, zur/yjbK*	None
	Plasmid pEc631_1	97,726	IncB/O/K/Z	None	*espI*	None	*traX, traV, traT, traS, traR, traQ, traO, traN, traM, traJ, traF, traE, traC, trbC, trbA, traW, traP*
	Plasmid pEc631_2	73,952	IncFII	None	None	None	*traM, traY, traA, traL, traE, traK, traB, traP, traV, traR, traC, traW, traU, traN, traF, traQ, traH, traG, traT, traD, traI, traX, trbB, trbC, trbE, trbF, trbI, trbJ*
	Plasmid pEc631_3	30,240	IncFII family	None	None	None	*trbM, trbG, trbI*
	Plasmid pEc631_4	7464	Col156	None	None	None	None
	Plasmid pEc631_5	5165	Col156	None	None	None	None
1500	Chromosome	4,736,377	–	None	*cfaA-E, ecpA-E, ecpR, elfA/G, elfC-D, eaeH, hcpA-C, fimA-I, ehaB, air/eaeX, upaG/ehaG, ibeB-C, sitA-D, espL1/L4/R1/X1, espX4–5,* ACE T6SS-like gene, *aec15–18, aec22–32, hlyE/clyA*	*cpxA/R, corA-D, nikA-E, nikR, cusB/F/S/X, soxR-S, emrA-B, emrD/K/R/Y, gadA-B, gadX, cutA/C, ars, arsC-B, arsR, dsbA-C, copA, tehA-B, modA-C, modE, ibpA-B, sodA/B, pstA-C, ptsS, marA/R, acrA/B, baeR/S, evgA/S, ydeO-P, bcr, cueO, fabI, glpF, iclR, mgtA, mntR, nfsA, oxyRkp, phoB, pitA, robA, rpoS, sugE, tolC, ychH, ydeI, ygiW, yhcN, yieF, yodD, ygjH, acrD/yffA, acrE/envC, acrF/envD, acrR/YbaH, actP/yjcG, bhsA/ycfR/comC, comR/ycfQ, cueR/ybbI, cusA/ybdE, cusC/ylcB, cusR/ylcA, cutE/Int, cutF/nlpE, emrE/mvrC, fetA/ybbL, fetB/ybbM, fieF/yiip, gadC/xasA, gadE/yhiE, gadW/yhiW, hdeA/yhiB, hdeB/yhiC, mdfA/cmr, mdtA/yegM, mdtB/yegN, mdtC/yegO, mdtE/yhiU, mdtF/yhiV, mdtG/yceE, mdtI/ydgE, mdtJ/ebrB/ydgF, mdtK/ydhE, mdtM/yjiO, mdtN/yjcR, mntH/yfeP, mntP/yebN, ostA/lptD, rcnA/yohM, rcnB/yohN, rcnR/yohL, ymgB/ariR, zinT/yodA, zitB/ybgR, zntA/yhhO, zntR/yhdM, znuA/yebL, znuB/yebI, znuC/yebM, zraR/hydH, zupT/ygiE, zur/yjbK*	None
	Plasmid pEc1500_CTX	91,123	IncI1	*bla*_CTX-M-15_, *bla*_TEM-1_	None	None	*traX, traV, traT, traS, traR, traQ, traP, traO, traN, traM, traJ, traE, traC, traA, traA, trbC, trbA, traW, traI*
	Plasmid pEc1500_2	4087	Col8282	None	None	None	None

^**α,β**^Details about the virulence genes and biocide/metal resistance genes are provided in Additional file 3, ^**γ**^Details about the conjugal transfer genes are provided in Additional file 4

### CTX-M-14 gene is located on the chromosome of *E. coli* strain 631

**Fig. 1:**
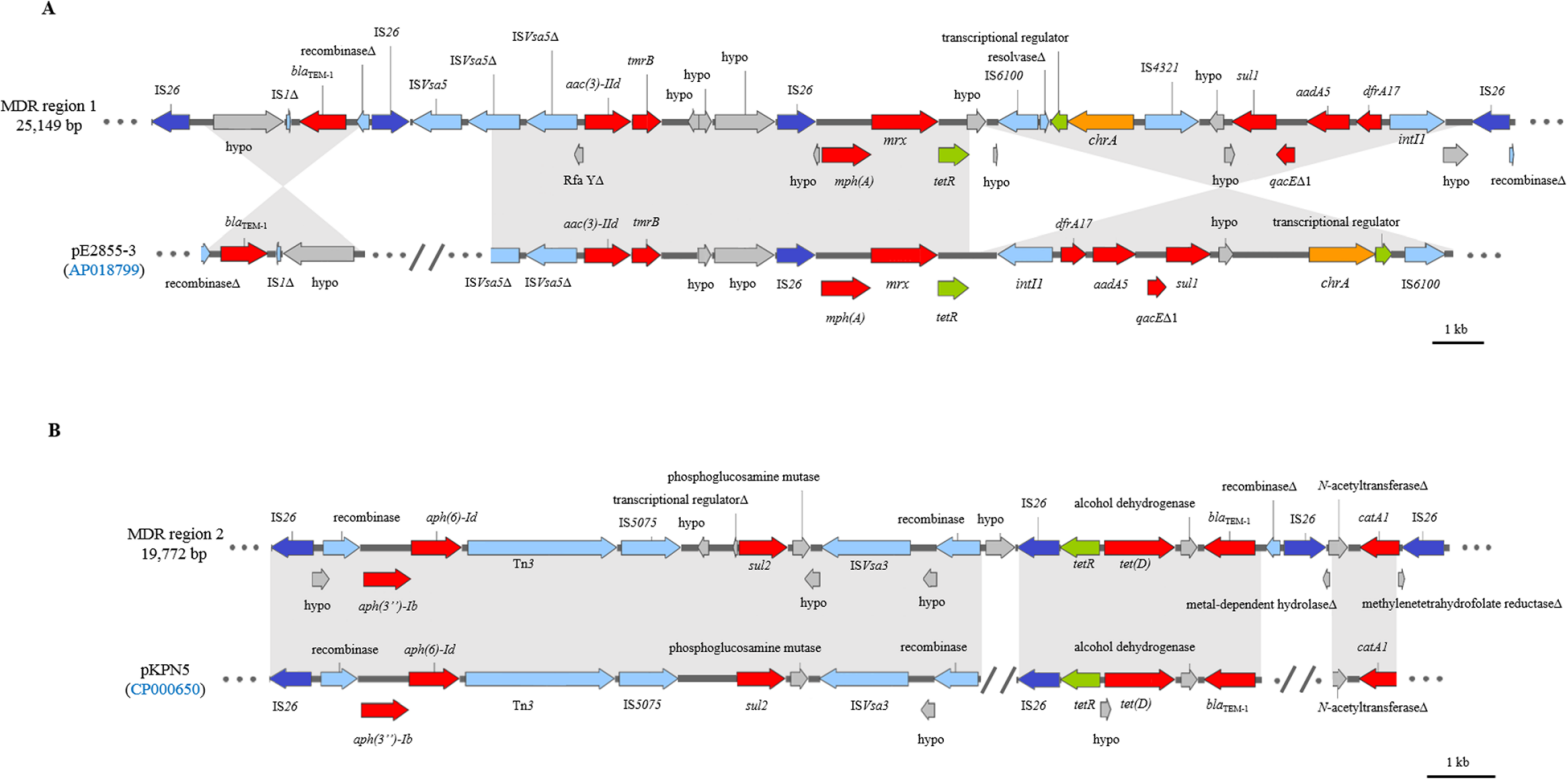
Map of chromosomal multidrug-resistance determining regions (MDR) in *Escherichia coli* strain 631. **a** MDR-1, located on the chromosome between positions 1,184,422 - 1,209,571 bp, flanked by IS*26* transposase, encoding *bla*_TEM-1_*, aac(3)-IId, tmrB, mph(A), mrx, sul1, qacE*Δ1*, aadA5* and *dfrA17*. **b** MDR-2, located on the chromosome between positions 4,406,649 - 4,426,421 bp, flanked by IS*26* transposase, encoding *aph(3″)-Ib, aph(6)-Id, sul2, tet(D), bla*_TEM-1_ and *catA1*. Arrows indicate the sizes of the ORFs and their orientations in the genome. Antibiotic resistance genes are highlighted in red, IS*26* transposases in dark blue, other transposases in blue, transcriptional regulators in green, metal resistance genes in orange and other genes are highlighted in dark grey. Δ represents truncated genes. Grey shaded regions represent > 99.9% nucleotide identity

Strain 631, belonging to ST38, carries all the ARGs on the chromosome (Table 1). The majority of ARGs are clustered together on the chromosome at two separate multidrug-resistance determining regions (MDRs), each flanked by IS*26* transposases on either end. MDR-1 (25,149 bp), located between positions 1,184,422 - 1,209,571 bp on the chromosome, carries genes conferring resistance to penicillins, tunicamycin, aminoglycosides, macrolides, sulfonamides and trimethoprim (Fig. 1a). This region harbors *bla*_TEM-1_, *tmrB*, *aac(3)-IId*, *aadA5*, *mph(A)*, *mrx*, *sul1*, *qacE*Δ1 and *dfrA17* genes. Additionally, MDR-1 carries a *chrA* gene, conferring chromate resistance [35]. MDR-2 (19,772 bp), located between positions 4,406,649 - 4,426,421 bp on the chromosome, carries genes conferring resistance to aminoglycosides, penicillins, amphenicols, tetracycline and sulfonamides (Fig. 1b). This region harbors *aph(3″)-Ib*, *aph(6)-Id*, *bla*_TEM-1_, *catA1*, *tet(D)* and *sul2* genes. Four identical copies of the *bla*_CTX-M-14_ gene are present on the chromosome of strain 631. Two of the *bla*_CTX-M-14_ copies are flanked by complete IS*5* and IS*Ec9* transposases, while the remaining two copies are flanked by a truncated IS*5* and a complete IS*Ec9* transposase.

**Fig. 2:**
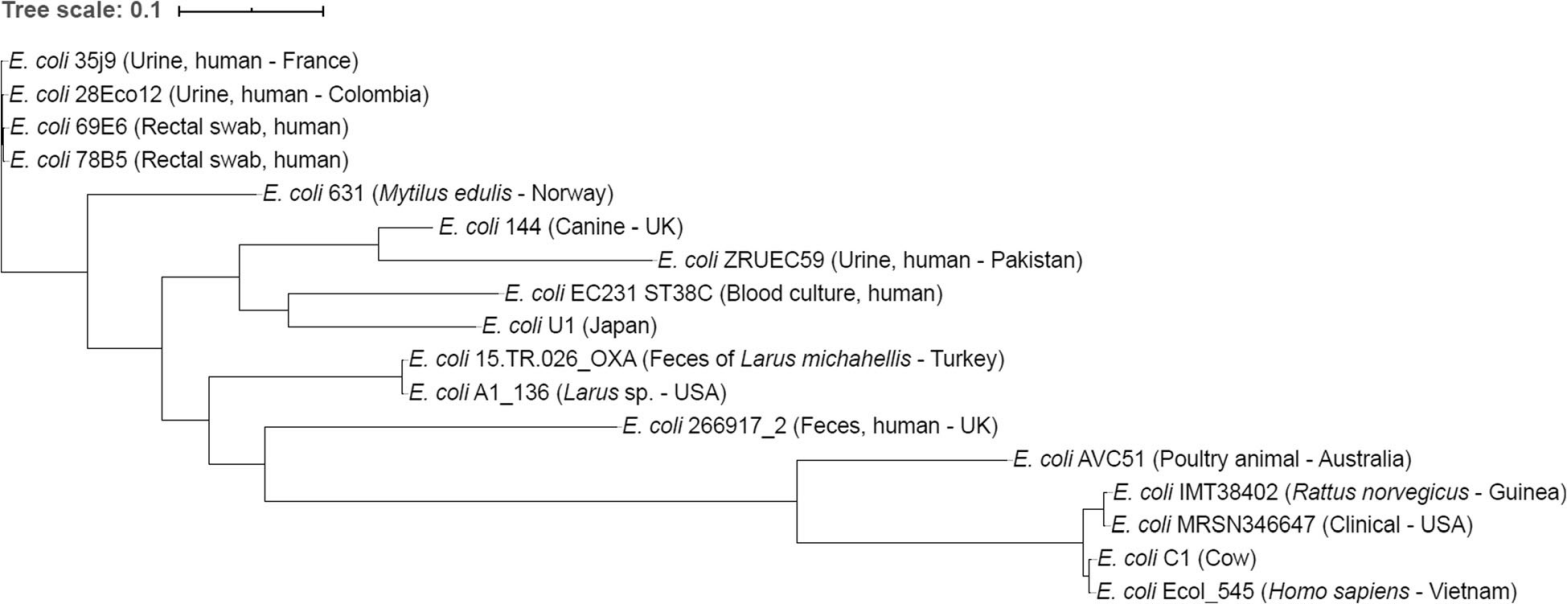
Single nucleotide polymorphism (SNP)-based phylogenetic tree of *Escherichia coli* strain 631 and genome sequences of other strains of ST38 retrieved from GenBank

A single nucleotide polymorphism (SNP)-based phylogenetic tree shows that *E. coli* strain 631 is clustering closer to human isolates, compared to ST38 isolates from other animals, suggesting a possible human origin of strain 631 (Fig. 2). The number of SNPs between strains 631 and other ST38 strains is presented in Additional file 5.

### *E. coli* strain 1500 carries CTX-M-15 gene on a conjugative IncI1 plasmid

**Fig. 3:**
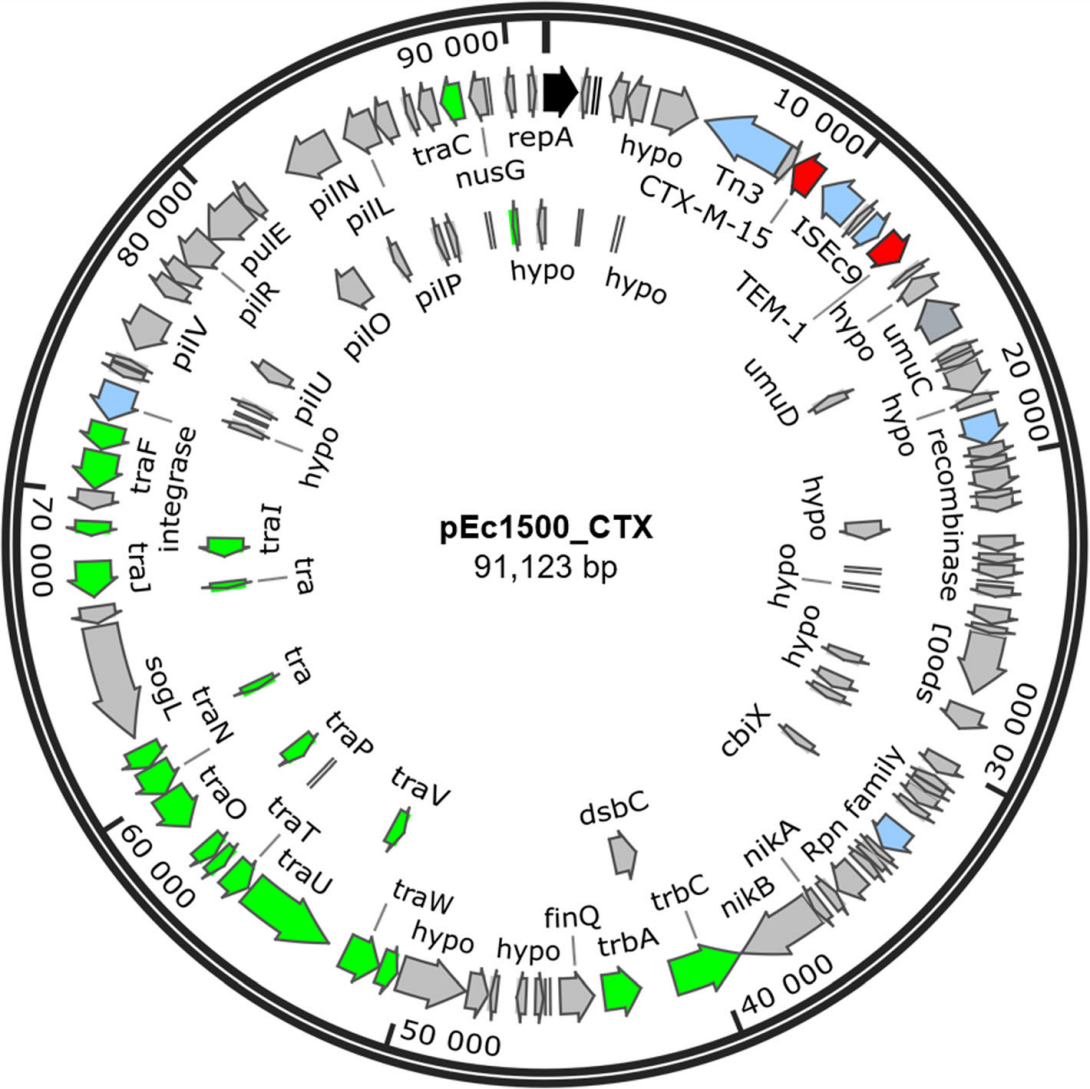
Structure of plasmid pEc1500_CTX carrying *bla*_CTX-M-15_ and *bla*_TEM-1_ genes (GenBank accession number: CP040270). Arrows indicate the sizes of the ORFs and their orientations in the genome. Antibiotic resistance genes are highlighted in red, transposases in blue, conjugal transfer genes in green, replication initiation gene in black and other genes are highlighted in grey

The CTX-M-15-encoding plasmid pEc1500_CTX belonging to IncI1 group (GenBank accession number: CP040270) is 91,123 bp and also carries *bla*_TEM-1_ gene (Table 1). The *bla*_CTX-M-15_ gene is located between positions 8445–9320 bp on the plasmid, flanked by Tn*3* and IS*Ec9* transposases (Fig. 3). The IS*Ec9* transposase flanking the *bla*_CTX-M-15_ gene in strain 1500 is identical (100%) to the IS*Ec9* transposase flanking *bla*_CTX-M-14_ in strain 631, further supporting the role of IS*Ec9* transposase in dissemination of CTX-M-type ESBLs [36].

## Discussion

To the best of our knowledge this is the first study reporting closed complete genome sequences of CTX-M-producing *E. coli* strains (631 and 1500) isolated from blue mussels (*Mytilus edulis*) in Norway. In accordance with previous studies, we used a combination of Nanopore and Illumina sequencing and hybrid *de novo* assembly combining Nanopore long-reads with the accuracy of Illumina reads, for obtaining closed complete genome sequences [37–41].

The multidrug-resistant *E. coli* strain 631 (ST38) was resistant to 15 antibiotics [27]. ST38 is a known pathogenic sequence type of *E. coli,* usually associated with intestinal disease and sometimes extra-intestinal infection [8]. Despite the number of plasmids harbored by this strain, all the ARGs are located on the chromosome clustered together at two separate MDRs, both flanked by IS*26* transposases. MDR-1 contains two DNA fragments (17,687 bp and 3094 bp, respectively) that are identical (> 99.9% nucleotide identity) to segments of a conjugative IncFII plasmid pE2855–3 (92.7 kb) reported in *E. coli* (GenBank accession number: AP018799) (Fig. 1a). MDR-1 also has DNA segments that are identical (> 99.9% nucleotide identity) to segments of a plasmid, pVPS43 (19.4 kb), reported in *Vibrio parahaemolyticus* (GenBank accession number: KX957970). MDR-2 contains three DNA fragments (13,222 bp, 4188 bp and 1176 bp, respectively) that are identical (> 99.9% nucleotide identity) to segments of plasmid pKPN5 (88.6 kb), reported in *Klebsiella pneumoniae* (GenBank accession number: CP000650) (Fig. 1b). High identity of MDRs to the segments of plasmids carried by known pathogens, indicate that these regions are potentially mobile. Strain 631 carried four identical copies of the *bla*_CTX-M-14_ gene on the chromosome, flanked by IS*Ec9* transposases. Our results are in accordance with previous studies reporting chromosomal CTX-M genes in *E. coli* ST38 [39, 42]. Although *bla*_CTX-M-14_ was detected on the chromosome of strain 631, the DNA fragment carrying *bla*_CTX-M-14_ and the flanking transposases, detected on the chromosome of strain 631, are identical (100%) to segments of plasmids carried by different members of the family *Enterobacteriaceae*, including *K. pneumoniae* (GenBank accession number: CP041102), *Salmonella enterica* (GenBank accession number: MH522424) and *Enterobacter cloacae* (GenBank accession number: CP035635), suggesting that *bla*_CTX-M-14_ carried by strain 631 is mobile*.*

IncFII is a well-known plasmid family contributing to the worldwide spread of clinically relevant ARGs, particularly *bla*_CTX-M-15_ [43]. We detected two IncFII plasmids in strain 631, which did not carry ARGs. Even though this is quite unusual, IncFII plasmids without ARGs have been reported previously [44–47]. Further, our analysis showed that the MDR-1 on the chromosome of strain 631 has DNA segments that are identical (> 99.9% nucleotide identity) to DNA segments of a conjugative IncFII plasmid reported in *E. coli* (GenBank accession number: AP018799) (Fig. 1a). This suggests a likelihood that the MDR regions in strain 631 may have been transferred from IncFII plasmid onto the chromosome by transposition [48].

*E. coli* strain 1500 carries CTX-M-15 gene on a conjugative IncI1 plasmid (pEc1500_CTX) that has high sequence identity (> 99.9%) with plasmid pSH4469 (91.1 kb), detected in CTX-M-15-producing *Shigella sonnei* (GenBank accession number: KJ406378) isolated from an outbreak in the Republic of Korea [49]. Plasmid pEc1500_CTX also has high identity (> 99.9%) with CTX-M-carrying plasmid pEK204 (93.7 kb) from an *E. coli* strain (GenBank accession number: EU935740) reported in the UK [50]. The plasmid backbone also shares high identity (> 99.9%) to a segment of ~ 61 kb from plasmid pHNRD174 (86.2 kb) from *E. coli* (GenBank accession number: KX246268) reported in China. Although CTX-M-14-encoding IncI1 plasmid has previously been reported in Norway [51], to the best of our knowledge, this is the first report on detection of *E. coli* carrying *bla*_CTX-M-15_ on an IncI1 plasmid in the marine environment in Norway. IncI1 plasmids are widely distributed within the family *Enterobacteriaceae* and are associated with dissemination of several ARGs [52]. The presence of CTX-M-15 gene on a conjugative IncI1 plasmid in strain 1500 [27] highlights the potential for transfer of CTX-M-15 to other environmental bacteria.

## Conclusion

This study highlights the usefulness of hybrid assembly combining accurate short-reads and long-reads for obtaining closed complete genome sequences of strains 631 and 1500. Thus, enhancing the understanding of the genomic arrangement and potential for mobility of clinically important ARGs. It demonstrates the potential role of the marine environment in dissemination of pathogenic *E. coli* strains and clinically relevant ESBLs. These observations strengthen the notion that the environment plays an important role in dissemination of clinically relevant ARGs and pathogens [13]. Our study also highlights the need for surveillance of antibiotic resistance in the environment, especially in a low prevalence setting like Norway, which would provide important insights for designing mitigation strategies for coping with resistance dissemination, before it becomes widespread.

## Methods

### Bacterial strains, DNA extraction and sequencing

*E. coli* strains 631 and 1500 were isolated from blue mussels (*M. edulis*) collected along the Norwegian coast, and characterized as described earlier [27]; the strains 631 and 1500 were denoted as strains B184 and B117, respectively, in Grevskott et al. 2017 [27]. *E. coli* strains 631 and 1500 were grown overnight on Mueller-Hinton (MH) agar (Oxoid, UK) containing 2 μg/mL cefotaxime sodium salt (Sigma-Aldrich, USA) at 35 °C. For Illumina sequencing, genomic DNA was extracted from the strains using the MagNA Pure 96 DNA Small Volume kit and a MagNA Pure 96 instrument (Roche Diagnostics, Germany). For Oxford Nanopore sequencing, the extraction and purification of high-molecular weight DNA was achieved, following the protocol described by Salvà-Serra et al. [53]. The DNA was quantified, using NanoDrop™ 2000 Spectrophotometer (Thermo Fisher, USA) assay and Qubit™ 2.0 Fluorometer with the dsDNA BR (Broad-Range) kit (Thermo Fisher, USA). Integrity of the DNA (i.e., > 60,000 bp) was verified, using a Genomic ScreenTape kit, on a 2200 TapeStation system (Agilent Technologies, Inc., USA).

For Illumina sequencing, Kapa HyperPlus Library Preparation kit (Kapa Biosystems, USA) was used to prepare sequencing libraries. Sequencing was performed on Illumina MiSeq platform (Illumina, USA), using 2 × 250 bp chemistry, at the Public Health Institute, Oslo, Norway. For Nanopore sequencing, the sequencing library was prepared, using a Rapid Barcoding kit (Oxford Nanopore Technologies Ltd., UK). The library was sequenced, using a MinION sequencer and a FLO-MIN 106D Flow Cell version R9.4.1 (Oxford Nanopore Technologies Ltd., UK).

### Genome assembly and sequence analysis

The raw reads generated by Illumina MiSeq were quality trimmed and assembled, using Trimmomatic version 0.36 [54] and SPAdes version 3.11.1 [55], respectively. The quality of the generated Illumina reads was analyzed with FastQC version 0.11.3 [56] and CLC Genomics Workbench version 12.0.3 (Qiagen, Denmark). The raw data generated by the MinION instrument were processed and demultiplexed with Guppy software version 2.3.7 (Oxford Nanopore Technologies Ltd.) and assembled using Canu version 1.8 [57]. The quality of the demultiplexed data was analyzed with NanoPlot version 1.26.3 [58].

Subsequently, a hybrid *de novo* assembly of Illumina and Nanopore reads was performed, using Unicycler version 0.4.7 [34]. Assembly statistics were obtained, using QUAST server [59]. Average Nucleotide Identity values based on BLAST (ANIb) [60] were calculated, using the server JSpeciesWS [61], between *E. coli* strains 631, 1500 and *E. coli* DSM 30083^T^ (GenBank accession number: AGSE00000000), to confirm the species identity. Genomes were annotated, using the Prokaryotic Genome Annotation Pipeline (PGAP) version 4.8 at the National Center for Biotechnology Information (NCBI) [62]. Complete overview of the genome sequences of strains 631 and 1500 were obtained, using GView Server version 1.7 [63]. Genetic maps were produced, using SnapGene® software version 4.3.8.1 (GSL Biotech, USA). Multi-locus sequencing types (MLSTs) were examined, using the MLSTs tool described by Larsen et al. [64], with *E. coli #1* MLST profile [65]. Plasmid replicons were typed, using PlasmidFinder 2.0 [66], as well as BLASTP analysis of the replication initiation (Rep) sequence against the NCBI database. The presence of ARGs was examined, using ResFinder 3.2 [67] and CARD 3.0.7 [68]. Virulence genes were analyzed, using the Virulence Factors Database (VFDB) [69], and BMRGs were examined, using the BacMet database 2.0 [70], using the script *BacMet-Scan.pl* against the database of “Experimentally confirmed resistance genes”. Conjugal transfer genes were examined by searching through the GenBank files of the annotated genome sequences of strains 631 and 1500.

### Comparative analysis of *E. coli* strain 631

A SNP-based comparative analysis of the *E. coli* strain 631 (ST38) with other strains of identical ST from different sources and countries was performed as described by Sabat et al. [71]. Briefly, the assembled genome sequences in FASTA format were analyzed, using the tool CSI Phylogeny 1.4 [72]. The parameters minimum depth at SNP positions, minimum relative depth at SNP positions, minimum distance between SNPs and minimum SNP quality were disabled, while the minimum read mapping quality and z-score were kept by default at 25 and 1.96, respectively. The SNP-based phylogenetic tree was displayed on-line with the Interactive Tree Of Life (iTOL) [73]. The details of the strains of *E. coli* ST38 included in the comparative analysis are presented in Additional file 6.

## Supplementary information

**Additional file 1.** Assembly statistics of complete genome sequences of strains 631 and 1500.

**Additional file 2. **Complete genome overview of CTX-M-producing *Escherichia coli* strains 631 (A) and 1500 (B).

**Additional file 3.** List of virulence genes and biocide/metal resistance genes detected in strains 631 and 1500 (i.e., names and their function).

**Additional file 4.** List of genes involved in conjugal transfer detected in strains 631 and 1500.

**Additional file 5. **Single nucleotide polymorphism differences in *Escherichia coli* strains 631 compared with *E. coli* ST38 strains from different sources and countries.

**Additional file 6. **List of *Escherichia coli* ST38 strains included in the single nucleotide polymorphism (SNP)-based analysis.

## Data Availability

The assembled genome sequences are submitted to GenBank under accession numbers: CP040263-CP040268 and CP040269-CP040271, respectively. Strains 631 and 1500 are available at the Culture Collection University of Gothenburg (CCUG; www.ccug.se) under the numbers CCUG 73937 and CCUG 73938, respectively.

## Abbreviations

AMR: Antimicrobial resistance
ANIb: Average nucleotide identity values based on BLAST
ARGs: Antibiotic resistance genes
BMRGs: Biocide/metal resistance genes
ESBL: Extended-spectrum β-lactamase
iTOL: Interactive Tree Of Life
MDRs: Multidrug-resistance determining regions
MH: Mueller-Hinton
MLSTs: Multi-locus sequence types
NCBI: National Center for Biotechnology Information
PGAP: Prokaryotic Genome Annotation Pipeline
SNP: Single nucleotide polymorphism
ST: Sequence type
VFDB: Virulence Factors Database
