# Supplementary material for: Nanopore sequencing reveals genomic map of CTX-M-type extended-spectrum β-lactamases carried by Escherichia coli strains isolated from blue mussels (Mytilus edulis) in Norway
Source: BMC Microbiol. 2020 May 25;20:134. doi: 10.1186/s12866-020-01821-8 (PMC7249450; doi:10.1186/s12866-020-01821-8)
Supplement: Supplementary file 1 — Additional file 1. Assembly statistics of complete genome sequences of strains 631 and 1500. [file 12866_2020_1821_MOESM1_ESM.docx]

**Additional file 1**. Assembly statistics of complete genome sequences of strains 631 and 1500.

| Strain | Genome size (bp) | GC content (%) | Genes (coding) | rRNA genes | tRNA genes | Illumina coverage | Oxford Nanopore coverage |
| --- | --- | --- | --- | --- | --- | --- | --- |
| 631 | 5,406,033 | 50.53 | 5,054 | 22 | 88 | 57 x | 670 x |
| 1500 | 4,831,587 | 50.72 | 4,481 | 22 | 85 | 121 x | 800 x |
