# Supplementary material for: Nanopore sequencing reveals genomic map of CTX-M-type extended-spectrum β-lactamases carried by Escherichia coli strains isolated from blue mussels (Mytilus edulis) in Norway
Source: BMC Microbiol. 2020 May 25;20:134. doi: 10.1186/s12866-020-01821-8 (PMC7249450; doi:10.1186/s12866-020-01821-8)
Supplement: Supplementary file 2 — Additional file 2. Complete genome overview of CTX-M-producing Escherichia coli strains 631 (A) and 1500 (B). [file 12866_2020_1821_MOESM2_ESM.pdf]

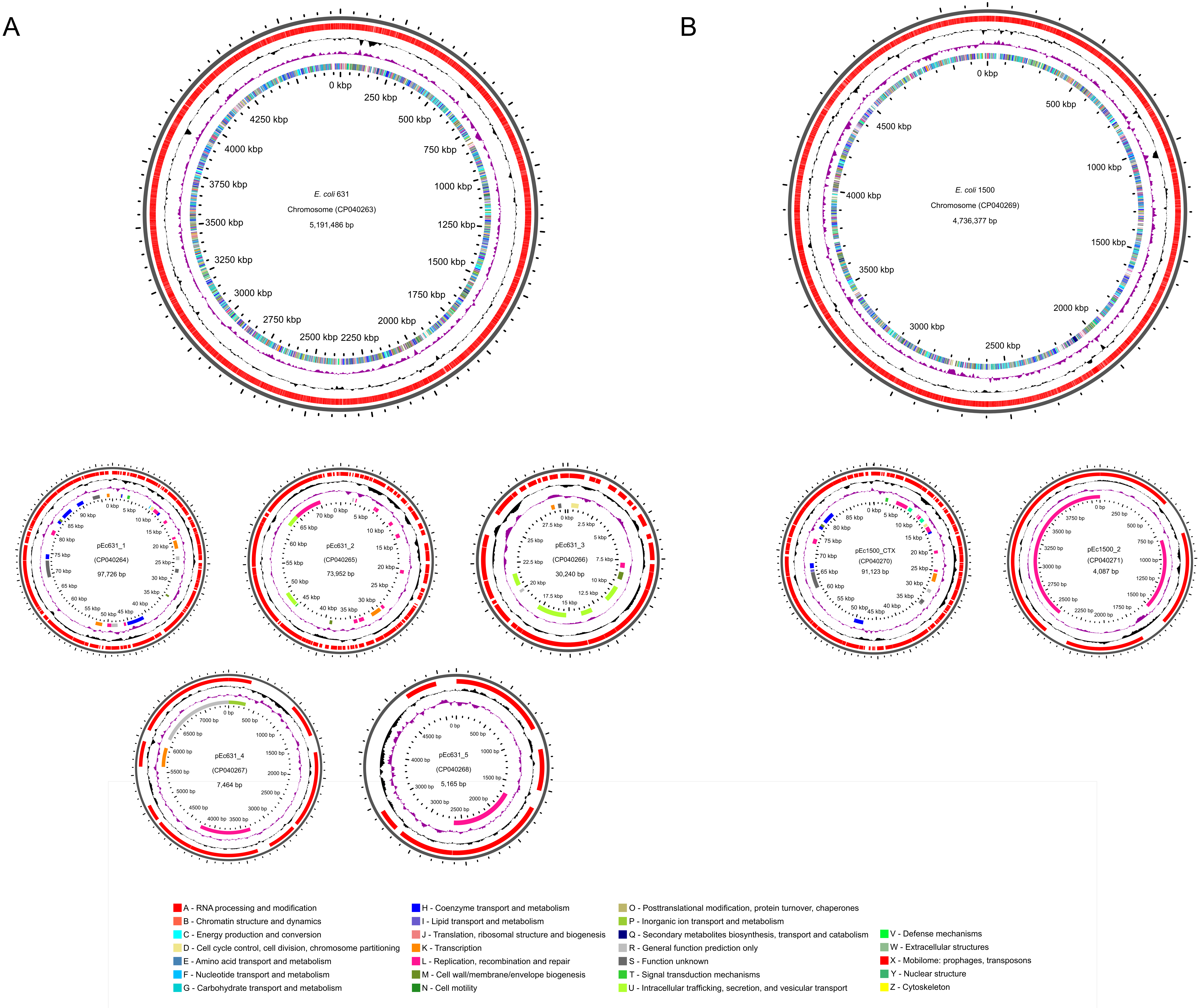
A
B
E. coli 631
Chromosome (CP040263)
5,191,486 bp
E. coli 1500
Chromosome (CP040269)
4,736,377 bp
pEc631_1
(CP040264)
97,726 bp
pEc631_2
(CP040265)
73,952 bp
pEc631_3
(CP040266)
30,240 bp
pEc1500_CTX
(CP040270)
91,123 bp
pEc1500_2
(CP040271)
4,087 bp
pEc631_4
(CP040267)
7,464 bp
pEc631_5
(CP040268)
5,165 bp
A - RNA processing and modification
B - Chromatin structure and dynamics
C - Energy production and conversion
D - Cell cycle control, cell division, chromosome partitioning
E - Amino acid transport and metabolism
F - Nucleotide transport and metabolism
G - Carbohydrate transport and metabolism
H - Coenzyme transport and metabolism
I - Lipid transport and metabolism
J - Translation, ribosomal structure and biogenesis
K - Transcription
L - Replication, recombination and repair
M - Cell wall/membrane/envelope biogenesis
N - Cell motility
O - Posttranslational modification, protein turnover, chaperones
P - Inorganic ion transport and metabolism
Q - Secondary metabolites biosynthesis, transport and catabolism
R - General function prediction only
S - Function unknown
T - Signal transduction mechanisms
U - Intracellular trafficking, secretion, and vesicular transport
V - Defense mechanisms
W - Extracellular structures
X - Mobilome: prophages, transposons
Y - Nuclear structure
Z - Cytoskeleton
