# Supplementary material for: Nanopore sequencing reveals genomic map of CTX-M-type extended-spectrum β-lactamases carried by Escherichia coli strains isolated from blue mussels (Mytilus edulis) in Norway
Source: BMC Microbiol. 2020 May 25;20:134. doi: 10.1186/s12866-020-01821-8 (PMC7249450; doi:10.1186/s12866-020-01821-8)
Supplement: Supplementary file 6 — Additional file 6. List of Escherichia coli ST38 strains included in the single nucleotide polymorphism (SNP)-based analysis. [file 12866_2020_1821_MOESM6_ESM.docx]

| **Organism** | **Strain** | **Genome sequence Accession nr.** | **Isolation source (host)** | **Country** |
| --- | --- | --- | --- | --- |
| *E. coli* ST38 | 266917_2 | CP026723 | Feces (human) | UK |
| *E. coli* ST38 | ZRUEC59 | QKMW00000000 | Urine (human) | Pakistan |
| *E. coli* ST38 | AVC51 | QOEC00000000 | Poultry animal (variety unknown) | Australia |
| *E. coli* ST38 | IMT38402 | PJMH00000000 | *Rattus norvegicus* (rat) | Guinea |
| *E. coli* ST38 | 28Eco12 | CP038505 | Urine (human) | Colombia |
| *E. coli* ST38 | 69E6 | MKGZ00000000 | Rectal swab (human) | Missing |
| *E. coli* ST38 | 78B5 | MKGT00000000 | Rectal swab (human) | Missing |
| *E. coli* ST38 | 35j9 | MKGX00000000 | Urine (human) | France |
| *E. coli* ST38 | EC231_ST38C | REBH00000000 | Blood culture (human) | Missing |
| *E. coli* ST38 | 15.TR.026_OXA | CP032145 | Feces of *Larus michahellis* (gull) | Turkey |
| *E. coli* ST38 | U1 | CP041359 | Missing | Japan |
| *E. coli* ST38 | A1_136 | CP040390 | *Larus* sp. (gull) | USA |
| *E. coli* ST38 | 144 | CP023364 | Canine | UK |
| *E. coli* ST38 | Ecol_545 | CP018976 | *Homo sapiens* | Vietnam |
| *E. coli* ST38 | C1 | CP010116 | Cow | Missing |
| *E. coli* ST38 | MRSN346647 | CP018206 | Clinical | USA |

**Additional file 6**. List of *Escherichia coli* ST38 strains included in the single nucleotide polymorphism (SNP)-based analysis.
